# The Salt Pack in Rheumatic Gout

**Published:** 1902-02-08

**Authors:** 


### THE SALT PACK IN RHEUMATIC GOUT.

Mr. Jonathan Hutchinson[1] says that he knows of no one remedy which is so almost universally effectual in getting rid of irritation and synovial effusion in connection with rheumatic gout than the salt pack. This consists of flannel soaked in a saturated brine of common salt, which is wrapped round the affected joint, covered with oiled silk and a bandage, and kept on the whole night. It should be applied every night until the cure is effected.

[1] The Polyclinic, January.

